# Molecular and Cellular Mechanisms of Neuroinflammation

**DOI:** 10.1155/2017/8417183

**Published:** 2017-12-03

**Authors:** Anna Di Vito, Giuseppe Donato, Daniele Tomassoni

**Affiliations:** ^1^Department of Clinical and Experimental Medicine, University Magna Graecia of Catanzaro, Catanzaro, Italy; ^2^Department of Health Science, University Magna Graecia of Catanzaro, Catanzaro, Italy; ^3^School of Biosciences and Veterinary Medicine, University of Camerino, Camerino, Italy

In the central nervous system (CNS), the innate immune response plays a significant role in both physiological and pathological conditions. CNS diseases including traumatic brain injury, ischemic stroke, brain tumor, and cerebrovascular and neurodegenerative diseases trigger a cascade of events broadly defined as neuroinflammation, which is characterized by the activation of the microglia and astrocyte population. On the other hand, microglial and astrocyte activation, T lymphocyte infiltration, and overproduction of inflammatory cytokines have been demonstrated in association with neuronal alteration in both animal and human tissues. Neuroinflammation is thus a hot topic in contemporary neuroscience. Further complicating the neuroinflammatory landscape is the fact that the immune-privileged status of the CNS is on trial, given the numerous studies showing that CNS is an actively regulated site of immune surveillance, as recently reviewed by Negi and Das [1]. A key role in the control of immune-surveillance is played by microglia, which, in addition to keeping the brain free from damaging insults, also contribute to neuronal functions with providing neurotrophic substances to regulate neurotransmitters and hormones and mediating responses to pain and stress challenges [2]. In this special issue, we have invited a few papers that address such important issues.

Recently, great attention has been directed to the mechanisms by which innate immune system might activate the endothelial cells of the blood-brain barrier (BBB) to arise neuroinflammation that eventually becomes unregulated. The paper of B. W. Festoff et al. provides interesting details about the role of BBB as well as damaged associated-molecular pattern (DAMPs) and coagulation factors in the onset and progression of neurodegenerative disorders.

The paper of L. F. Hernández-Zimbrón et al. focused the attention on the activation of immunological responses in aged brain, which contribute to the development of neurodegenerative disease. The paper showed the accumulation of the amyloid-*β* peptide 1–42 (A*β*42), glial fibrillary acidic protein (GFAP), and presenilin 2, hallmarks of Alzheimer's disease, in endothelial cells, blood vessels, and neurons of the visual cortex in aged mice.

In the preclinical study, A. P. Herman et al. highlighted the importance of the interaction between the immunity and the neuroendocrine system. Acute and prolonged inflammation account for the release of proinflammatory cytokines, which ultimately interfere with the secretion of gonadotropin-releasing hormone (GnRH) and luteinising hormone (LH) altering the normal estrus. One possible strategy to block the inflammatory status is the stimulation of acetylcholine (ACh) secretion or the inhibition of acetylcholinesterase (AChE) activity. A. P. Herman et al. showed the possibility of blocking inflammatory-dependent changes in the GnRH/LH secretion via the administration of AChE inhibitors which did not pass the BBB, without interfering in the CNS.

Neuroinflammation is recognized as one of the potential mechanisms mediating the onset of a broad range of psychiatric disorders. The review of F. A. Radtke et al. provided a clear overview of the molecular mechanism underlying neuroinflammation in mental illness, such as schizophrenia, bipolar disorder, depression, anxiety, obsessive-compulsive disorder, and autism. An interesting interaction between mental disorders-associated copy number variants and inflammation was proposed.

G. Zhang and P. Yang explained the molecular mechanisms involved in the establishment of chronic neuropathic pain associated with spinal cord injury and suggested the chemokine Ccl3 and the MAP kinase signaling pathway as potential therapeutic targets to alleviate neuropathic pain.

The paper of R. Maldonado-Ruiz et al. focused the attention on signaling pathways involved in the process of metabolic inflammation in obesity and its modulation by neuropeptides such as POMC-derived peptides, ghrelin, and leptin. In the CNS, neuropeptides modulate inflammation and migration of peripheral cells into the CNS via the BBB and may represent a molecular node during positive energy balance as is the obesity and maternal overnutrition.

The papers of this special issue provided important insights into CNS-peripheral immune system dialogue, highlighting the role of molecular factors and signaling pathways in the onset and progression of different neurological diseases.

*Anna Di Vito* *Giuseppe Donato* *Daniele Tomassoni*
